# Making proteomics data accessible and reusable: Current state of proteomics databases and repositories

**DOI:** 10.1002/pmic.201400302

**Published:** 2015-03-12

**Authors:** Yasset Perez-Riverol, Emanuele Alpi, Rui Wang, Henning Hermjakob, Juan Antonio Vizcaíno

**Affiliations:** European Molecular Biology Laboratory, European Bioinformatics Institute (EMBL-EBI), Wellcome Trust Genome CampusHinxton, Cambridge, UK

**Keywords:** Bioinformatics, Databases, MS, Repositories

## Abstract

Compared to other data-intensive disciplines such as genomics, public deposition and storage of MS-based proteomics, data are still less developed due to, among other reasons, the inherent complexity of the data and the variety of data types and experimental workflows. In order to address this need, several public repositories for MS proteomics experiments have been developed, each with different purposes in mind. The most established resources are the Global Proteome Machine Database (GPMDB), PeptideAtlas, and the PRIDE database. Additionally, there are other useful (in many cases recently developed) resources such as ProteomicsDB, Mass Spectrometry Interactive Virtual Environment (MassIVE), Chorus, MaxQB, PeptideAtlas SRM Experiment Library (PASSEL), Model Organism Protein Expression Database (MOPED), and the Human Proteinpedia. In addition, the ProteomeXchange consortium has been recently developed to enable better integration of public repositories and the coordinated sharing of proteomics information, maximizing its benefit to the scientific community. Here, we will review each of the major proteomics resources independently and some tools that enable the integration, mining and reuse of the data. We will also discuss some of the major challenges and current pitfalls in the integration and sharing of the data.

## 1 Introduction

In the age of systems biology and data integration, proteomics data represent a crucial component to understand the “whole picture” of life. Proteomics technologies—particularly MS-based protein identification and quantification approaches—have matured immensely through cumulative advances in high-throughput analytical methodologies [1–5], sample preparation [6], improved instrumentation [7], and the availability of protein sequence databases [8] and computational analysis tools [1,9]. Therefore, with the development of more powerful and sensitive analytical methods and instrumentation, the identification and quantification of a high proportion of the expressed proteins in a given condition is now achievable in an average experiment [10,11]. In parallel, as a result, the size of data produced in proteomics laboratories has increased by several orders of magnitude [12].

Compared to other data-intensive fields such as genomics, deposition and storage of original proteomics, data in public resources have been less common [13]. This is regrettable since proteome studies are usually more complex than its counterpart genomics ones. In fact, data interpretation in proteomics can be considerably more complex than in genomics due to the wide variety of analytical approaches [14,15], bioinformatics tools and pipelines [16,17], and the related statistical analysis [18,19]. However, thanks to the guidelines promoted by several scientific journals and funding agencies [20], there is a growing consensus in the community about the need for the public dissemination of proteomics data, which is already facilitating the assessment, reuse, comparative analyses, and extraction of new findings from published data [13,21].

The complexity of proteomics data is heightened by alternative splicing, PTMs, and protein degradation events, and is further amplified by the interconnectivity of proteins into complexes and signaling networks that are highly divergent in time and space [1]. In order to address this complexity, new analytical and bioinformatics methodologies are developed every year [22,23], which complicate the data standardization and deposition. Additionally, the audience interested in proteomics data is very heterogeneous. It includes, biologists elucidating the mechanisms of regulation of specific proteins, MS researchers improving the current analytical methods, or computational biologists developing new software tools for the analysis and interpretation of the data [24].

Data sharing in proteomics requires substantial investment and infrastructure. Several public repositories have been developed, each with different purposes in mind. Well-established databases for proteomics data are the Global Proteome Machine Database (GPMDB) [25], PeptideAtlas [26], and the PRIDE database [27]. Additionally, at present there are other resources (many of them recently developed) such as ProteomicsDB [28], MassIVE (Mass Spectrometry Interactive Virtual Environment), Chorus, MaxQB [29], PASSEL (PeptideAtlas SRM Experiment Library) [30], MOPED (Model Organism Protein Expression Database) [31], PaxDb [32], Human Proteinpedia [33], and the human proteome map (HPM) [34]. Furthermore, there are several more specialized resources that will only be cited briefly in this review. It is important to mention here that no single proteomics data resource will be ideally suited to all possible use cases and all potential users. Regrettably, two widely used resources were discontinued due to lack of funding: Peptidome [35] and Tranche [36]. This had a negative impact on the efforts promoting data sharing in the field, as it was perceived by the community that effort invested in data sharing was lost.

Recently, the ProteomeXchange (PX) consortium [37] has been formed to enable a better integration of public repositories, maximizing its benefits to the scientific community through the implementation of standardized submission and dissemination pipelines for proteomics information. By August 2014, PRIDE, PeptideAtlas, PASSEL, and MassIVE are the active members of the consortium.

The aim of this review is to provide an up-to-date overview of the current state of proteomics data repositories and databases, providing a solid starting point for those who want to perform data submission and/or data mining. There are a few comparable reviews available in the literature [24,38–41], but there is a need for an update since this has been quite a dynamic field over the past few years. In this manuscript, we will not include a thorough review about protein knowledge bases, such as the Universal Protein resource (UniProt) [42] and neXtProt [43], but we will explain how MS proteomics information is made available in these resources.

## 2 Organization of proteomics repositories and databases

The information generated in a typical proteomics experiment can be organized in three different levels [44]: (i) raw data; (ii) processed results, including peptide/protein identification and quantification values; and (iii) the resulting biological conclusions. Technical and/or biological metadata can be provided for each level independently.

**Figure 1:**
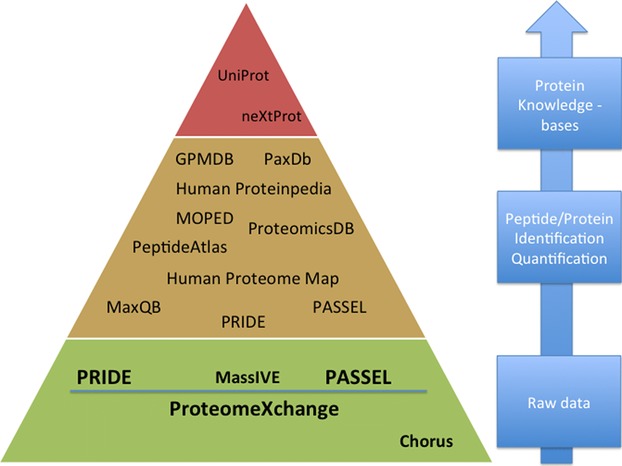
Hierarchy of proteomics data repositories and databases according to the different data types stored: raw MS data repositories, resources that store peptide/protein identification and quantification results, and protein knowledge bases. Some resources are duplicated in different levels because they can be included in more than one category.

These three categories enable the classification of the existing MS proteomics repositories according to their level of specialization (Fig.1). In our view, these three levels of information should be captured and properly annotated in public databases and repositories, ideally using data standards, when available. In fact, the development of proteomics resources is more feasible due the maturity of some data standard formats [45–47] and open source tools [9], which facilitate public data deposition.

Some of the first open formats developed included mzXML (for MS data) [48], pepXML, and protXML (for peptide/protein identifications) that were developed as part of the trans-proteomic pipeline (TPP) [49]. In the context of the Proteomics Standards Initiative (PSI), several standard data formats have been developed over the last few years, which reflect the variety in data types within the field, and therefore those that can be supported by proteomics resources. The main formats developed have been mzML (for MS data) [45], mzIdentML (for peptide and protein identification results) [46], mzQuantML (for capturing a detailed trace of each stage of quantitative analysis) [47], TraML (for representing input transitions in SRM approaches) [50], and the recent mzTab (for capturing a simpler summary of the final results) [51]. The aims and functionalities of the existing resources will be explored in detail in the following sections (Table1).

**Table 1:** Main characteristics of the major MS-based proteomics repositories and databases

Repositories	Raw data	Support for targeted approaches	Metadata	Human protein expression information	Species	Quantification data	Related stand-alone tools	Web services URL	URL
PRIDE (June 2014)	X	–	High level	41 835 Protein accessions 269 806 Unique peptide sequences Approximately 101 million spectra	Approximately 450 species	X	PRIDE Inspector, PRIDE Converter 2, PeptideShaker	http://www.ebi.ac.uk/pride/ws/archive/	http://www.ebi.ac.uk/pride/
PeptideAtlas (Human, August 2013)	X	X	Medium level	14 018 Proteins 338 013 Peptides Approximately 258 million spectra	*Mus musculus, Candida albicans, Candida*, *Caenorhabditis elegans, Drosophila melanogaster, Halobacterium, Equuscaballus, Rattus norvegicus, Saccharomycescerevisiae, Danio rerio, Susscrofa, Mycobacterium tuberculosis*	–	TIQAM, TIQAM–Digestor, TIQAM–PeptideAtlas, TIQAM–Viewer, ATAQS, PIPE2, PABST		http://www.peptideatlas.org/
GPMDB (May 2014)	–	X	High level	136 373 Protein accessions 1 786 698 Peptides Approximately 1020 million spectra	*Bos taurus, Canis familiaris, Homo sapiens, M. musculus, R. norvegicus, Gallus gallus, D. rerio, Xenopus tropicalis, Anopheles gambiae, Apis mellifera, D. melanogaster, C. elegans, Oryza sativa, Arabidopsis thaliana, Saccharomyces cerevisiae, Bacillus anthracis* Ames, *Escherichia coli* (K12), *Lactococcus lactis* (Il1403), *M. tuberculosis, Shigella dysenteriae, Salmonella typhi, Salmonella typhimurium* (LT2*)*	–	–	http://rest.thegpm.org/1	http://gpmdb.thegpm.org/
MassIVE (May 2014)	X	–	Low level	^*^	^*^	–	–	–	http://massive.ucsd.edu/
Chorus (May 2014)	X	–	Low level	^*^	^*^	–	–	–	https://chorusproject.org/
ProteomicsDB (May 2014)	X	–	High level	18 097 Proteins 739 406 Peptides Approximately 70 million spectra	*H. sapiens*	X	–	–	https://www.proteomicsdb.org/
MOPED (May 2014)	–	–	Medium level	17 141 Proteins 250 000 Unique peptides Approximately 15 million spectra	*H. sapiens*, *M. musculus*, *C. elegans*, *S. cerevisiae*	X	–	–	https://www.proteinspire.org/MOPED/
Human Proteinpedia (May 2014)	X	–	Low level	15 231 Proteins 1 960 352 Peptides Approximately 5 million spectra	*H. sapiens*	–	–	–	http://www.humanproteinpedia.org/
MaxQB (May 2014)	–	–	Medium level	14 732 Proteins 370 551 Peptides Approximately 20 million spectra	*M. musculus*, *H. sapiens* *S. cerevisiae*	X	–	–	http://maxqb.biochem.mpg.de/mxdb/
PaxDb (May 2014)	–	–	Low level	10 482 Proteins 143 456 Peptides Approximately 24 million spectra	*A. thaliana, M. musculus, H. sapiens, S. cerevisiae, D. melanogaster, E. coli* (K12), *Microcystis aeruginosa, C. elegans, Leptospira interrogans* serovar *Copenhageni, M. tuberculosis* (H37Rv), *Streptococcus pyogenes M1 GAS, Schizosaccharomyces pombe, B. taurus, S. dysenteriae, B. subtilis, G. gallus, Thermococcus gammatolerans* (EJ3), *Mycoplasma pneumoniae, Halobacterium* (NRC–1), *S. typhimurium* LT2, *R. norvegicus, Deinococcus deserti* (VCD115), *Shigella flexneri, Synechocystis, A. mellifera, C. familiaris, Sus scrofa, X. tropicalis, O. sativa*	X	–	http://pax-db.org/api/search?q=human	http://pax-db.org/
HPM (June 2014)	–	–	Low level	10 482 Proteins 293 000 Unique peptides Approximately 25 million spectra	*H. sapiens*	X	–	–	http://humanproteomemap.org

X, the feature or characteristic is supported; -, the feature is not supported; ^*^, it was not possible to retrieve the corresponding information.

## 3 Resources

### 3.1 The PX consortium

The PX consortium [37] (http://www.proteomexchange.org) was created to promote the collaboration and integration of major stakeholders in the domain of MS proteomics repositories comprising, among others, primary (PRIDE [27] and PASSEL [30]) and secondary resources (PeptideAtlas), proteomics researchers, and representatives from journals regularly publishing proteomics data. Recently, in June 2014, MassIVE joined the consortium. The aim of PX is to provide a common framework for the cooperation of proteomics resources by defining and implementing consistent, harmonized, user-friendly data deposition and dissemination procedures. In addition, another important goal is to enable and provide “mutual backup” if one of the resources has funding issues.

The consortium's members have agreed in providing a sufficient set of common experimental and technical metadata. This information is stored using the PX XML format [37]. Finally, all the submitted datasets get a unique and universal identifier (PXD identifier).

#### 3.1.1 Data submission and format support

By August 2014, two major workflows are fully supported in PX: MS/MS and SRM approaches. In the first stable implementation of the data workflow, PRIDE acts as the initial submission point for MS/MS data, whereas PASSEL has the equivalent role for SRM data. Both workflows will be explained in detail below. At the moment of writing, MassIVE has just joined PX aiming to have an equivalent role to PRIDE.

There are two different PX MS/MS submission modes: “Complete” and “Partial.” To perform a “complete” submission means that after all the files have been submitted, it is possible for the receiving repository to connect directly the processed identification results with the mass spectra. This can be achieved if the processed identification results are available in a format supported by the receiving repository (e.g., mzIdentML) and if peak list files are included in the submission. “Complete” submissions get a Digital Object Identifier (DOI) to facilitate its traceability.

On the other hand, after performing a “partial” submission the connection between the spectra and the identification results cannot be done in a straightforward way. In this case, the processed results are not available in a supported format by the receiving repository and the corresponding search engine output files (in heterogeneous formats) are made available for download. For both types of submissions, metadata and raw data are always stored for each dataset.

Although “partial” submissions are searchable by their metadata, peptide and protein identifications cannot be captured by the receiving repository, which decreases the ability of reviewers to check the data and can make data reuse by third parties challenging. For instance, “partial” submissions do not qualify for the requirements on spectra annotation from the journal MCP (*Molecular and Cellular Proteomics*—http://www.mcponline.org/site/misc/ParisReport_Final.xhtml).

Finally, it needs to be highlighted that all PX members support private review of the data during the manuscript review process. The submitted data remains private before manuscript publication and login details are provided to facilitate access for reviewers and journal editors during the manuscript review process.

#### 3.1.2 Data mining and visualization

ProteomeCentral (http://proteomecentral.proteomexchange.org) is the centralized portal for accessing all PX datasets, independently from the original resource where data were stored. ProteomeCentral provides the ability to search metadata associated with datasets in the participating repositories (PRIDE and MassIVE for MS/MS data, PASSEL for SRM data, or reprocessed original PX datasets in PeptideAtlas). It is then possible to query the archive and identify datasets of interest using biological and technical metadata, keywords, tags, or publication information. To monitor the release of new public PX datasets, researchers can subscribe to a Rich Site Summary feed (http://groups.google.com/group/proteomexchange/feed/rss_v2_0_msgs.xml). Next, the main characteristics of the individual members of the consortium will be explained.

### 3.2 PRIDE

**Figure 2:**
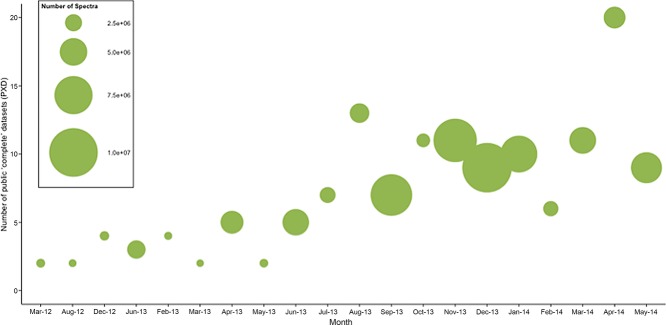
Bubble chart representation of the size of the PX complete submissions to PRIDE (until May 2014). The *x*-axis includes months with at least one submission, since PX submissions started (from March 2012). The *y*-axis corresponds to the number of PX “complete” public datasets submitted to PRIDE in each specific month. The size of each bubble represents the total number of mass spectra included in all the datasets in a given month.

The PRIDE database [27] (http://www.ebi.ac.uk/pride/) was initially developed at the European Bioinformatics Institute (EBI, Cambridge, UK) to store the experimental data included in publications, supporting the manuscript review process. The main data types stored in PRIDE are peptide/protein identifications (including PTMs), peptide/protein expression values, the analyzed mass spectra (both as raw data and peak lists), and the related technical/biological metadata. In PRIDE, data are stored as originally analyzed by the researchers (the author's analysis view on the data), supporting many popular search engines/analysis workflows. Most of the terms, information and metadata supporting the PRIDE data, are based on ontologies or controlled vocabularies [52]. In this context, the “Ontology Lookup Service” [53] was developed as a spin-off of PRIDE to enable the querying, browsing, and navigation of biomedical ontologies. Since the inception of PX, the number of datasets submitted to PRIDE has grown considerably (Fig.2). In parallel, the size of the experiments in terms of spectrum numbers has also increased significantly (Fig.2).

#### 3.2.1 Data submission and format support

As a member of the PX consortium, PRIDE supports both “complete” and “partial” submissions. For “complete” submissions, processed identification results need to be provided in PRIDE XML (PRIDE original data format) or the PSI standard mzIdentML (version 1.1) format. If mzIdentML is used, the corresponding peak list files referenced by the mzIdentML files need to be included as well. A “complete” submission ensures that the processed results data can be integrated in PRIDE and visualized using tools, such as PRIDE Inspector [54] (see below). Therefore, for performing a “complete” submission, the output files from the analysis software need to be converted or exported to either mzIdentML (see http://www.psidev.info/tools-implementing-mzidentml and http://www.ebi.ac.uk/pride/help/archive/submission/mzidentml) or PRIDE XML (using PRIDE Converter 2 [55] or other external tools). It is important to highlight that the recently implemented support for mzIdentML makes possible that data from a growing number of tools/analysis software (that were never supported via PRIDE XML) can now be fully supported by PRIDE.

The PRIDE Converter 2 tool suite (http://pride-converter-2.googlecode.com) [55] is an open source and platform-independent software that allows users to convert several search engine output files to PRIDE XML. The tool suite consists of four different applications: PRIDE Converter 2, PRIDE mzTab generator, PRIDE XML merger, and PRIDE XML filter. All of these tools can be launched using the graphical user interface or from the command line. PRIDE Converter 2 currently supports the output from MASCOT (.dat) [56], X!Tandem (.xml) [57], OMSSA (.csv), among others. It also supports different mass spectra file formats (mzML, dta, mgf, mzData, mzXML, and pkl). As mentioned above, there are other tools not developed by the PRIDE team that can also export to PRIDE XML [37]. It is expected that, as the mzIdentML format becomes more and more popular, the use of PRIDE XML will decrease in time.

The “partial” submission route is aimed at situations where processed identification results cannot be generated in mzIdentML/PRIDE XML if there is no converter/exporter available. However, the “partial” submission mechanism also enables any data from any proteomics workflow to be stored in PRIDE. As a consequence, some datasets are already available coming from workflows, such as top-down proteomics, MS imaging, or SWATH-MS, among others.

The PRIDE/PX submission process has been recently described in detail [58]. The PX submission tool (http://www.proteomexchange.org/submission) [37] is an open-source standalone tool that provides a user-friendly graphical user interface for performing the actual data submission, through a series of steps:

- Select all the files needed for submission.
- Interactively group-related different types of files (e.g., the corresponding raw and processed results files).
- Ensure a minimum level of metadata (according to the PX guidelines).
- Transfer the files via the Aspera (from version 2.1) or FTP file transfer protocols. Aspera (http://asperasoft.com/) can perform up to 50 times faster than FTP, enabling a convenient way of transferring large datasets.

In addition to the PX submission tool, datasets containing a high number of files can also be submitted using a command line based alternative (http://www.ebi.ac.uk/pride/help/archive/aspera) [58]. Each dataset becomes publicly available on acceptance or publication of the corresponding manuscript, or when the authors tell PRIDE to do so.

#### 3.2.2 Data mining and visualization

PRIDE Inspector (http://pride-toolsuite.googlecode.com) [54] is an open source standalone tool that can be used to efficiently browse and visualize MS proteomics data. PRIDE Inspector can be used by researchers before submission and also by journal editors and reviewers during the manuscript review process. The latest version available at the moment of writing (version 2.1) supports identification results in PRIDE XML and mzIdentML (used in PX “complete” submissions). It also supports spectra files in a variety of formats (mgf, pkl, ms2, mzXML, mzData, and mzML). PRIDE Inspector enables users to visualize and check the data at different levels. It has different panels devoted to experimental metadata, protein, peptide, and spectrum-centric information. Finally, the “summary charts” tab provides eight different charts that can be used to evaluate some aspects of the quality of the dataset. Apart from the visualization functionality, it can access some of the most popular protein databases (UniProt knowledgebase (UniProtKB), Ensembl [59], and the National Center for Biotechnology Information (NCBI) nonredundant database) to retrieve the most up-to-date protein sequences and names for the reported protein identifiers.

The PRIDE Archive website (http://www.ebi.ac.uk/pride/archive/) provides the web interface to query and retrieve the information in PRIDE. It was launched on January 2014 and at the moment of writing, is still under iterative development, so new features are being added constantly. By August 2014, the current version allows querying PRIDE using keywords, publication, species, tissues, diseases, modifications, instruments, peptide sequences, and protein identifiers. When a specific dataset is selected, the users are directed to the dataset summary page, which also lists the assays (equivalent to the old PRIDE experiment numbers) related to the project, in the case of “complete” submissions. Users can download all the files via FTP or Aspera, and/or visualize the results using PRIDE Inspector. In the coming months, visualization for peptide/protein identifications will be available in the PRIDE web. At present the BioMart interface (http://www.ebi.ac.uk/pride/legacy/prideMart.do) is the easiest way to access this information. However, it is planned that it will be soon replaced by new PRIDE web services.

Additionally, PRIDE data can be accessed using the “PRIDE Cluster” webpage (http://www.ebi.ac.uk/pride/cluster/) [60]. It includes public identified spectra in PRIDE that have been clustered using the “PRIDE Cluster” algorithm (https://code.google.com/p/pride-spectra-clustering/). The PRIDE Cluster resource currently provides two main methods for accessing its data: (i) retrieve all clusters that contain a given peptide identification and (ii) retrieve all clusters with a consensus spectrum similar to a queried spectrum. In addition, spectral libraries for several species are also provided. “PRIDE Cluster” is the first quality control step attempted in a highly heterogeneous MS proteomics repository, such as PRIDE.

### 3.3 PASSEL

PASSEL [30] (http://www.peptideatlas.org/passel/) supports the submission of datasets generated by SRM approaches by storing the experimental results and the corresponding raw data. The submitted raw data are automatically reprocessed in a uniform manner using mQuest, a component of the mProphet software suite [61], and the results are loaded into the database [62]. The original files and the corresponding reprocessed results are made available to the community. Additionally, the measured transitions are incorporated into the SRMAtlas [62] catalog of transitions. Detailed metadata and structured sample information are in accordance with the PX guidelines.

#### 3.3.1 Data submission and format support

PASSEL uses a web interface for the data submission process (http://www.peptideatlas.org/submit) and is now supporting the following data types (http://www.peptideatlas.org/upload/): (i) study metadata, such as dataset title, submitter contact information, sample source, sample preparation, and instrument used; (ii) transition lists describing which transitions were measured for each peptide and targeted ions, along with optional supporting information (collision energy, expected retention time, and expected relative intensities). This information is available in a tab-separated file or in the standard TraML format [50]; and (iii) mass spectrometer output files in mzML [45] or mzXML [48] format. If these are not available, the vendor formats.wiff (AB SCIEX),.raw (Thermo), or.d (Agilent) are also supported. All the files supplied by the users are uploaded to a specially created FTP account and they can be browsed using the “PASSEL Experiment Browser”.

#### 3.3.2 Data mining and visualization

The “PASSEL Experiment Browser” enables filtering based on fields, such as research contact information, organism, sample, and instrument type. As mentioned above, it is also possible to download the original files provided (http://db.systemsbiology.net/sbeams/cgi/PeptideAtlas/GetSELExperiments).

The “PASSEL Data Browser” provides a description of each selected transition group and the data collected for it, a link to visualize the trace group using the “Chromavis” chromatogram viewer and further links to the information available in SRMAtlas [62] and PeptideAtlas [26], in order to extract or compare spectral features of the targeted peptides (http://db.systemsbiology.net/sbeams/cgi/PeptideAtlas/GetSELTransitions).

### 3.4 PeptideAtlas

The PeptideAtlas project (http://www.peptideatlas.org) [26,63,64] was originally created to serve as the end-point for the TPP processing software [49]. In recent years, PeptideAtlas has grown as a data reprocessing resource and it has served as a research database for the development of spectral libraries [65] and SRM-related tools [66,67]. For instance, PeptideAtlas provides information about proteotypic peptides using detectability scores [68].

Nowadays, PeptideAtlas is one of the biggest and well-curated protein expression data resources. The initial PeptideAtlas publication reported the identification of 27% of the human genes in Ensembl with a protein false discovery rate (FDR) likely 10% or higher [63]. Over the years, the PeptideAtlas team has developed different tools to control the assignment of incorrect identifications, such as PeptideProphet [69] and ProteinProphet [70], and more recently MAYU [71], to control the protein FDR when different datasets are combined. This platform and the statistically accurate protocol used to curate the protein/peptide identification data have turned PeptideAtlas into a very reliable protein expression database. In 2013, the generated 1% protein-level FDR Human PeptideAtlas had at least one peptide for around 14 000 different UniProtKB/Swiss-Prot entries [26].

In the context of PX, partners have recently started to track the reanalysis of original PX datasets by providing RPXD identifiers to those, and linking them to the original reprocessed datasets. By August 2014, several PX datasets reanalyzed by PeptideAtlas are already publicly available in ProteomeCentral.

#### 3.4.1 Data submission and format support

PeptideAtlas reprocessed data are organized into different builds [72], each includes data from a single proteome or subproteome (see species in Table1). Each build is generated with raw MS/MS spectra submitted using the “submission form” (http://www.peptideatlas.org/upload/) or the data deposited into another public repository, such as PRIDE. These spectra are searched against a sequence database, a spectral library or both. Peptide and protein identifications are mapped to a comprehensive reference protein database (for the latest human builds, the searched database is a combination of UniProtKB/Swiss-Prot, Ensembl, and sequences from the International Protein Index (IPI)), and postprocessed using the TPP [72]. It also annotates each protein and peptide with supporting data, such as genome mappings, sequence alignments, links to different databases, such as GPMDB or the Human Protein Atlas [73], uniqueness of peptide–protein mappings, observability of peptides, predicted observable peptides, estimated protein abundances and cross-references to other databases, such as RefSeq, UniGene and UniProt. All the processed results are loaded into SBEAMS (systems biology experiment analysis management system) proteomics that is a proteomics analysis database built as a module under the SBEAMS framework.

#### 3.4.2 Data mining and visualization

The PeptideAtlas web search interface can be used to search for proteins by protein accession, peptide sequence, gene name, keyword, or phrase. If a specific protein is requested, the “protein view page” summarizes all the information available for that protein. The top section provides basic information about the protein, including alternative names as well as the total number of corresponding spectra (observations) and distinct peptides. The following two sections, “sequence motifs” and “sequence,” summarize the peptide coverage of the protein. Finally, a similar diagram to a genome browser view summarizes all the peptides that map either uniquely or redundantly to the protein, including information on segments unlikely to be observed by MS. Information about signal peptides and transmembrane domains is also provided, where available. One of the best features of PeptideAtlas is the Cytoscape [74] plug-in that allows the user to view the distinct peptides for a particular protein as a network with associated proteins.

Recently, PeptideAtlas implemented the “PeptideAtlas Chromosome Explorer” (http://www.peptideatlas.org/peptideatlasExplorer/) to summarize and classify the identified human proteome using a chromosome-oriented view. The present version shows the number of protein observations and the UniProt entries by chromosome using a circular histogram plot. PeptideAtlas is actively involved in the HUPO chromosome-based Human Proteome Project (C-HPP) [75] and provides a dedicated website to access protein identifications per chromosome (http://www.peptideatlas.org/hupo/c-hpp/).

PeptideAtlas heavily supports targeted proteomics workflows in different ways: (i) SRMAtlas (http://www.srmatlas.org/) is a compendium of targeted proteomics assays to detect and quantify proteins using SRM/MRM-based proteomics workflows; (ii) TIQAM (targeted identification for quantitative analysis by MRM) [76], which is a desktop application to facilitate the selection of peptide and transitions. It consists of three applications: “TIQAM-Digestor,” “TIQAM-PeptideAtlas,” and “TIQAM-Viewer”; (iii) the automated and targeted analysis with quantitative SRM tool (ATAQS) [77] is a software pipeline tool that contains modules to design, manage, analyze, and validate an MRM assay. ATAQS uses FireGoose [78] to connect to various web services, such as PeptideAtlas (used to select spectra), TIQAM (to generate in silico peptides for a given protein [76]), PIPE2 (to generate a list of proteins, to design an MRM assay, and for other various analysis tasks), and PABST (peptide atlas best SRM transition, used to generate optimal transitions).

### 3.5 MassIVE

The MassIVE data repository (http://massive.ucsd.edu) is a community resource developed by the Center for Computational Mass Spectrometry (University of California, San Diego) to promote the global-free exchange of MS data. MassIVE provides a location for researchers to access public raw datasets and accompanying files, often alongside publication. One of the key features of MassIVE (still under development) compared with other resources is that its functionality is aimed at networking and providing a social platform for researchers. MassIVE users are not only able to browse, download, but also comment on datasets. These comments can be accompanied with new data or new analyses that enrich the original dataset.

#### 3.5.1 Data submission and format support

The submission to MassIVE is based on two main steps: (i) upload the data files to ProteoSAFe (http://massive.ucsd.edu/ProteoSAFe/); and (ii) invoke the MassIVE dataset submission workflow for those files. The MassIVE team strongly recommends that submitters use FTP to upload and organize their dataset files as opposed to the ProteoSAFe file upload web interface.

MassIVE dataset files are organized into the following categories: (i) license files—specifying how and under which conditions the dataset files may be downloaded and used; (ii) spectrum files—any mass spectrum files constituting the main body of a dataset (in mzXML and/or raw binary format); (iii) result files—the output of any search engine; (iv) sequence databases—any protein or other sequence databases that were searched against (.fasta format); (v) spectral libraries—any spectral library files that were searched, if applicable; (vi) methods and protocols, for any open-format files containing explanations or discussions of the experimental procedures used to obtain or analyze a given dataset.

The MassIVE submission web presents an input form including all the files to be uploaded and relevant metadata. The metadata fields include the species, instruments, PTMs, and contact information. When a dataset is submitted, the ProteoSAFe validation is performed.

#### 3.5.2 Data mining and visualization

The “MassIVE datasets” browser (http://massive.ucsd.edu/ProteoSAFe/datasets.jsp) presents a list of all public datasets in a tabular format. Users can sort and filter by any column. Alternatively, users can select specific datasets by checking the boxes next to them. MassIVE tried to rescue as many datasets as possible from the defunct Tranche repository. For this imported data, the Tranche hash is also displayed, enabling the users to search for specific Tranche datasets.

Once data are submitted to the repository, it can be shared with others (e.g., reviewers) using password-protected access to the datasets, or be made publicly available by the submitter. In the near future, it is planned that anyone with access to a given dataset will be able to reanalyze it using the many workflows available in ProteoSAFe. After the reanalysis, the results can then be used to either enrich existing datasets or to create new ones. MassIVE enables on-site reanalysis using a variety of data workflows: standard database searches (MS-GF+) [79], proteogenomics searches against genomics/transcriptomics sequences (ENOSI) [80–82], discovery of unexpected modifications (MODa) [83], identification of mixture spectra using spectral library (MSPLIT) [84] and database (MixDB) search [85], de novo sequencing of peptides (PepNovo) [86] and proteins (Meta-SPS) [87], molecular spectral networks (including both peptides and metabolites), and top-down protein identification (MS-Align+) [88].

### 3.6 Chorus

Chorus (http://chorusproject.org) is a cloud-based application that provides scientists the ability to store, analyze, and share their MS data regardless of the original raw file format generated. The Chorus team's aim is to create a complete catalogue of the mass spectrometric data in a way that can be openly accessed, and make it freely accessible to both the scientific community and the general public. Chorus was originally announced in 2013 and is enabled by Amazon Web Services, Inc. (http://aws.amazon.com). It is a global cloud-based computing environment that provides customized data analysis tools that convert proprietary data formats to a common “Map Reduce” format for processing large datasets using parallel and distributed algorithms on the cloud. The initial data analysis tools include chromatographic and mass spectral viewers as well as a database search engine for protein sequence identification.

The registered users should first request laboratory membership or create their own laboratory. After that, they can create a project, an instrument, and upload raw files. Once this is done, the users can create experiments and organize them into the created projects.

### 3.7 GPMDB

GPMDB [25] (http://gpmdb.thegpm.org) is one of the most well-known protein expression databases. The GPMDB pipeline reprocesses the MS/MS data provided by users or raw data stored in other repositories, such as PX, using the popular open source search engine X!Tandem [57]. Peptide and protein identifications are generated and stored in XML files, which are indexed in a MySQL database.

#### 3.7.1 Data submission and format support

The data submission process supports MS data in different formats (.dta,.pkl,.mgf, mzXML, and mzData) and it can take place via the “simple search page.” Once the data have been processed using X!Tandem, users can choose whether to submit their data or not to GPMDB. In addition to X!Tandem, users can also use a spectral search engine called X!Hunter (http://xhunter.thegpm.org/) [89] or the proteotypic peptide profiler X!P3 (http://p3.thegpm.org/) [90] to analyze their data. All the identified peptides are matched to the Ensembl [59] genome database.

#### 3.7.2 Data mining and visualization

The GPMDB main entry point is a web-based search interface, where users can search by keywords, data sources, protein identifiers, or gene names. Also, the current GPMDB implementation provides another three alternative pages to quickly access and search the data: (i) dataset search by accession (also called gpm #, http://gpmdb.thegpm.org/gpmnum.html); (ii) sequence search (also called sequence, http://gpmdb.thegpm.org/seq.html); (iii) access based on GO terms [91], chromosome, Kyoto Encyclopedia of Genes and Genomes (KEGG) pathways [92], BRaunschweig ENzyme DAtabase (BRENDA) [93] tissue ontology (http://gpmdb.thegpm.org/go/index.html); and (iv) the amino acid polymorphisms and PTMs search for proteins.

When a search is performed, the main “protein table” lists all the matched proteins and different features are shown, such as protein accession, number of observations in GPMDB, log(e), and an evidence code that rates the current evidence for the observation of each protein. The “protein view” shows all the peptide identifications for a specific protein and the corresponding sequence coverage to specify the reliability of the identification.

More recently, GPMDB has been actively involved in the C-HPP project [94]. In this context, human protein identification information in GPMDB is now summarized into a collection of spreadsheets called “Guide to the Human Proteome.” This guide contains the information organized into separate spreadsheets for each chromosome as well as for the mitochondrial DNA. It also contains protein accession numbers, HGNC (HUGO (Human Genome Organization) Genome Nomenclature Committee) [95] gene names, and chromosomal coordinates taken from Emsembl.

### 3.8 ProteomicsDB

ProteomicsDB (http://www.proteomicsdb.org/) is a human protein expression database that stores protein and peptide identifications and quantification values. The resource was jointly developed by the Technical University of Munich, the company SAP (Walldorf, Germany), and the SAP Innovation Center. It was announced in 2013 and it has been recently highlighted as the main output of a study drafting the human proteome [28]. It contains information of more than 62 projects and more than 300 experiments. The proteins identified in the resource map to over 18 000 human genes, representing around 90% of the human proteome. However, the FDR calculations when different datasets are combined were not taken into account in the analysis [18], as PeptideAtlas does [63,71]. It currently contains approximately 70 million spectra from human cancer cell lines, tissues, and body fluids. ProteomicsDB enables real-time analysis and is based on the SAP HANA platform (http://www.saphana.com) for rapid data mining and visualization. It has been built to enable public sharing of datasets as well as to enable users to access and review data prior to publication.

#### 3.8.1 Data submission and format support

The submission pipeline is well structured in three different levels: (i) projects that contain the general information, such as publication, title, and an experiment summary; (ii) experiments that include a name, description, and scope (the scope represents a definition of the experiment type, e.g., PTM or full tissue proteome); and (iii) experiment files—when the users upload a set of files, each file is sent to a verification process before it is definitely accepted or rejected. A large number of the experiments in the database were downloaded from repositories such as Tranche, PRIDE/PX, and PeptideAtlas [37], and later reprocessed using two parallel pipelines based on Andromeda/MaxQuant [96] and MASCOT, and quantified using MaxQuant [97].

#### 3.8.2 Data mining and visualization

A user-friendly web interface allows users browse the human proteome, including protein-level information, such as protein function and expression. Protein expression can be visualized across the complete human body. This novel feature is integrated into the “Protein expression tab” and comprises the human body map showing the expression of a given protein in more than 30 tissues, organs and body fluids. Also, the protein expression of cell lines can be projected onto the tissue of origin and experimental details for any sample of interest can be readily obtained. The “chromosome view” shows those proteins identified in each chromosome region, including the description of the proteins, length, unique peptides, unique PSMs (peptide spectrum match), shared PSMs and sequence coverage.

### 3.9 MaxQB

MaxQB (http://maxqb.biochem.mpg.de/mxdb/) [29] was released in 2012 as a database that stores and displays collections of large proteomics projects and allows joint analysis and comparison. MaxQB serves as a generic repository and analysis platform for high-resolution bottom-up experiments. It stores details about protein and peptide identifications together with the corresponding high- or low-resolution fragment spectra and quantitative information, such as protein ratios or label-free derived intensities.

#### 3.9.1 Data submission and format support

Differently to other databases, MaxQB only stores experiment data generated locally by the Mann group. This feature reduces greatly the data heterogeneity and facilitates the data analysis. To enable smooth upload of data, MaxQB is tightly integrated with MaxQuant [97]. When a MaxQuant analysis is performed, the user of MaxQuant is asked whether she/he wants to upload the data or not. This new feature will allow the integration of new datasets from others into MaxQB. Alternatively, the data can be manually uploaded through the web interface.

Additional metadata information should be provided in the submission process, such as the project name, experiment name, and workflow parameters. All the data are stored in a relational database. Several human protein sequence databases (UniProt including variants, Ensembl and IPI) were uploaded to MaxQB to build a reference protein database.

#### 3.9.2 Data mining and visualization

The MaxQB web interface allows the users query the database by different fields, such as gene name, organism, and source database. Alternatively, an advance query builder can be used if the user is not familiar with the query syntax. Apart of protein detail information and sequence coverage, MaxQB can also display expression information within any of the proteomes compared with all other quantified proteins in that proteome. The expression of a given protein is estimated by the sum of its peptide signals after normalization of the total proteome signals to each other in MaxQuant. The iBAQ algorithm [98,99], based on spectral counting, is now implemented in MaxQuant and can also be used to estimate protein quantification values. One of the major conclusions that MaxQB data analysis provides is that the peptide rank order can be used as a component of the protein identification score.

### 3.10 MOPED

MOPED (http://moped.proteinspire.org) [31,100] is an expanding resource that enables rapid browsing of protein expression information coming from humans and several model organisms. MOPED provides protein-level expression data, meta-analysis capabilities, and quantitative data from standard analyses. In order to address the metadata diversity, the MOPED database developed a multi-“omics” metadata checklist that has been used for collecting metadata, which has been made available to the community through DELSA (http://www.delsaglobal.org).

#### 3.10.1 Data submission and format support

In order to start a dataset submission, MOPED requires a minimum set of metadata that must be included at the experiment level. Users must then supply a brief experimental description, the source organism, and the journal reference. The MOPED pipeline [101] is based on reanalyzing MS data from other public repositories using SPIRE (Systematic Protein Investigative Research Environment http://proteinspire.org) [102]. SPIRE integrates open source search tools, such X!Tandem, OMSSA, and various statistical models into its pipeline. MOPED estimates protein absolute expression and concentration values using spectral counting. Since the probability of identifying peptides depends on peptide properties, such as the peptide sequence, the APEX quantitative approach weights spectral counts using estimates of these probabilities to improve the accuracy of the provided absolute expression values [101]. Each identified protein links to various protein and pathway databases, including GeneCards [103], UniProt, KEGG and Reactome [104]. The current MOPED database contains data from four of the most studied organisms: human, mouse, *Caenorhabditis elegans* and *Saccharomyces cerevisiae*.

#### 3.10.2 Data mining and visualization

The web interface contains three main panels: “protein absolute expression” “protein relative expression” and “gene relative expression.” Each of the panels contains the “MOPED search box” that supports queries by keywords, tissues, conditions, and pathways. After a search is performed, the “protein ID and expression summary” section displays expression data. Each protein row in the expression summary table displays the protein accession, description, concentration, organism, localization, sequence coverage, spectral count, and gene name.

The interface also features a “visualization panel” known as the chord diagram. It can break down proteins in experiments by organism, tissue, localization, and condition. The absolute and relative expression matrix in these panels shows the expression of the identified proteins per condition. In the future, MOPED plans to integrate proteomics with transcriptomics and metabolomics data through biological pathways and networks.

### 3.11 PaxDb

The first version of the PaxDb resource (http://pax-db.org/) [32] was published and released in 2012. PaxDb is a database dedicated to integrate information on absolute protein abundance levels, based on a deep coverage of the proteome, consistent data postprocessing, and enabling comparability across different organisms. PaxDb is a meta-resource since it takes the information previously published in data repositories, such as PRIDE and PeptideAtlas, and does not accept any direct data submissions.

#### 3.11.1 Data submission and format support

The current PaxDb pipeline analyzes the spectral counting information from PeptideAtlas builds—that is, which peptides have been identified and how often over the whole build. This part of PaxDb's data import is entirely based on the original scoring and quality cutoffs implemented by PeptideAtlas. In the case of identified peptide sequences reported from MS/MS approaches (e.g., PRIDE datasets), the current pipeline remaps each peptide to the corresponding protein, based on sequence matches using reference genomes from the STRING database [105]. Protein abundance values are converted for each dataset into protein abundance estimates, using a consistent value across different techniques. Instead of using “molar concentration” or “molecules per cell,” the system expresses all abundances in “parts per million.” This means that each protein is listed relatively to all other protein molecules in the same sample. In the case of biochemical, biophysical, or label-free MS experiments, parts per million values are directly computed by rescaling the provided abundance estimates by their total sum. In the case of spectral counting data, the method is based on the likelihood of detectability, as described earlier [106].

#### 3.11.2 Data mining and visualization

The PaxDb website allows an ad hoc query of a protein family of interest, allowing multiple proteins requests simultaneously, as well as browsing and comparing complete datasets. In addition, the user queries are searched against the annotations of all proteins in PaxDb using a full-text search. For each organism, a distinct summary page provides information on the data origin, its coverage and estimated quality, and the distribution of abundance values of each dataset as a histogram. Additionally, the most abundant proteins in the organism are also listed. The PaxDb protein view shows a brief description of the protein functional role as annotated by UniProt and/or by other model organism databases. The species browser allows the navigation through all the proteins for a specific model organism. Furthermore, it shows all the protein abundances in a specific dataset and also for the whole of the PaxDb datasets (addition of all datasets for a given specific species). All PaxDb data are freely available.

### 3.12 Human Proteinpedia

Human Proteinpedia (http://www.humanproteinpedia.org) [33] is a public human proteome repository for sharing protein expression data derived from multiple experimental platforms. It incorporates diverse features of the human proteome, including protein–protein interactions, enzyme–substrate relationships, PTMs, subcellular localization, and expression of proteins in various human tissues and cell lines, in diverse biological conditions (including disease states). The Human Proteinpedia database falls neatly in the annotation-oriented database category [38]. It complements the curated human protein reference database (HPRD) [107] with community-provided annotation. It collects submitted proteomics data and uses the findings to directly annotate the protein entries in HPRD with observed PTMs and subcellular localization [108]. In contrast with the resources previously described, Human Proteinpedia does not only contain MS-based experiments, but also other approaches such as coimmunoprecipitation, Western blotting, fluorescence, immunohistochemistry, and protein and peptide microarrays. All the data should be annotated by the users (http://pdas.hprd.org/) and it is not reprocessed.

#### 3.12.1 Data submission and format support

Users can provide data annotations in four different ways, after registering in the system: (i) data on individual proteins along with experimental evidence through the use of web forms, (ii) upload data via the web in a batch mode, (iii) sending data through FTP/e-mail to the support team, and (iv) distributed annotation service servers setup by the contributing laboratories for the data upload [109]. By June 2014, the current version includes data from more than 249 laboratories, including 2710 distinct experiments, more than 15 000 proteins, almost 2 million peptides, around 5 million spectra, and 2906 annotations related to subcellular localization (Table1).

The major differences with other repositories are as follows: (i) it does not exclusively contain MS-derived data, as mentioned already; (ii) data from proteomics experiments are viewed in the context of a protein–protein interaction resource (HPRD); (iii) it restricts the data to that derived from human tissues or cell lines; and (iv) data annotation related to various protein features can be done manually.

#### 3.12.2 Data mining and visualization

The query page in Human Proteinpedia (http://www.humanproteinpedia.org/query) enables the search by gene symbol, protein name, accession number, type of protein feature, and type of experimental platform annotated. After a query, the “protein detail view” includes a table where each row shows a “platform” (the type of the experiment, e.g., MS or immunohistochemistry) and related metadata. In the case of MS experiments, each platform contains a table containing all the peptides identified for each protein. All the data are freely available (http://www.humanproteinpedia.org/download).

### 3.13 HPM

The HPM (http://www.humanproteomemap.org) [34] has just been developed as an output of a recent draft study of the human proteome. The original proteomics study [34] was carried out on 30 histologically normal human tissues and primary cells using high-resolution MS. The generated tandem mass spectra correspond to proteins encoded by 17 294 genes, accounting for approximately 84% of the annotated protein-coding genes in the human genome. However, FDR calculations when different datasets are combined are not discussed in enough detail in the original manuscript [18]. The aim of the HPM is to make possible to review, navigate and visualize the protein expression information evidences of gene families, protein complexes, signaling pathways and biomarkers.

#### 3.13.1 Data submission and format support

MS/MS data obtained from all different experiments were searched against the Human RefSeq database using SEQUEST [110] and MASCOT [56] through the Proteome Discoverer platform (Thermo Fisher Scientific). Then, *q* values were estimated using the Percolator algorithm within the Proteome Discoverer suite. Protein and peptide identifications obtained from SEQUEST and MASCOT were converted into MySQL tables. NCBI RefSeq annotations were used as additional information about the genes from various public resources. Normalized spectral counts [34] were used to represent expression of proteins and peptides. The resource was developed as a protein expression database and do not support the submission of new data from external users.

#### 3.13.2 Data mining and visualization

The web portal was developed using a three-tier web architecture with presentation, application, and persistence layers. Users are able to search using gene or protein identifiers and the information is presented in a tabular format. For each peptide, a high-resolution MS/MS spectrum from the best scoring identification is shown on the spectrum viewer page using the “Lorikeet” JQuery plugin (https://code.google.com/p/lorikeet).

### 3.14 Other proteomics resources

There are other less widely used resources that will not be explained here in detail. First of all, the Cardiac Organellar Protein Atlas Knowledgebase (COPaKB; http://www.heartproteome.org/copa/) [111] is a centralized platform of high-quality cardiac proteomics data, bioinformatics tools, and relevant cardiovascular phenotypes. Currently, COPaKB features eight organellar modules, comprising 4203 MS/MS experiments from human, mouse, *Drosophila* and *C. elegans* as well as expression data of 10 924 proteins in the human myocardium. COPaKB has an attractive web interface which provides a number of effective workflows to guide cardiovascular investigators from the actual proteomics data to the systematic biomedical interpretation. The COPaKB team continues to develop innovative workflows to help providing a better understanding of protein functions in cardiovascular diseases. COPaKB will cover additional modules on organelles and cells from cardiovascular-relevant model systems. The content of each module will expand with the available public data.

Pep2pro (http://fgcz-pep2pro.uzh.ch/) [112,113] is a comprehensive analysis database specifically suitable for performing flexible data analysis. Pep2pro is a further development of the “AtProteome” resource and provides data from *Arabidopsis thaliana*. The current pipeline employs PepSplice [114] and TPP tools for peptide/protein identification, the characterization of whole genome hits and the PTMs. The database is organized in assemblies, similarly to the PeptideAtlas builds. The *Arabidopsis* assembly from 2011 contained more than 14 522 protein identifications, 141 235 identified peptides, and around 2 million spectra.

iProX (Integrated proteome resources, http://www.iprox.org/) is a repository jointly developed by Beijing Proteome Research Center and other institutions in China. It has been recently developed reusing part of the PRIDE source code. At the moment of writing, it is not fully operational but contains already some stored datasets.

### 3.15 Proteomics information available through UniProt and neXtProt

UniProt (http://www.uniprot.org) [115] is among the most used of the protein sequence and functional annotation providers. Among the UniProt databases is the UniProtKB, which provides a broad range of protein sequence datasets for a large number of species, specifically tailored for an effective coverage of the sequence space while maintaining a high-quality level of sequence annotations and mappings to the genomics and proteomics information.

MS proteomics data deposited in the main public repositories is flowing into UniProtKB to enrich protein sequence annotations at the level of the evidence, supporting the existence of a protein (isoforms and variant-containing sequences included). This information is thus provided to users mainly in two different ways: (i) indirectly via the UniProtKB protein existence values (http://www.uniprot.org/manual/protein_existence) that are starting to be assigned also on the PSM-level content publicly provided by proteomics repositories (together with the accompanying statistical assessment for each PSM) and (ii) directly through explicit links to relevant cross-referenced resources (http://www.uniprot.org/database), which cover PRIDE, PeptideAtlas, MaxQB and PaxDb, but also others such as PhosphoSitePlus [116].

Organism-specific mappings of the peptides reported in the repositories to the UniProtKB sequences are also shared with groups producing genome builds to improve the corresponding gene annotations. In the future, it is planned that PTMs [117] from proteomics repositories will also be integrated in UniProtKB.

neXtProt (http://www.nextprot.org) [43] is a web-based protein knowledge platform to support research uniquely on human proteins. The set of manually curated annotations extracted from UniProtKB/Swiss-Prot for human, which constitutes the heart of the resource, is constantly complemented with quality-filtered carefully selected high-throughput experiments from different scientific research areas concerning abundance, distribution, subcellular localization, interactions, and cellular functions. neXtProt is part of the consortium, which is driving the C-HPP project. Thus, neXtProt labels as “missing proteins” those ones, which so far lack experimental evidence [43].

All the relevant proteomics MS-related information (like for instance PTM-related MS-based papers on *N*-glycosylation, phosphorylation, S-nitrosylation, ubiquitination, and sumoylation) has been integrated into neXtProt and is available via the web interface in the “Proteomics view” of the “Protein perspective” layout.

## 4 Data reuse from public resources

Since the current volume of proteomics data deposition is rapidly increasing, new approaches based on the reanalysis of the data and/or new uses of the stored data are being developed. The same public dataset can be analyzed using different pipelines to discover, confirm, or highlight new biological evidences. Resources such as GPMDB and PeptideAtlas have been doing this for many years already, emphasizing control of the number of false-positives at both peptide and protein level.

Data availability per se in repositories has enabled data reanalysis by third parties triggering a discussion in the field about controversial datasets [118–122]. Furthermore, targeted reanalysis of public data by individual groups is now starting to flourish. Remarkably, the draft of the human proteome reported recently and available in ProteomicsDB includes a big proportion of reanalyzed public datasets (roughly 40% of the MS runs) [28]. In addition, new PTMs have also been described as a result of reanalysis of public datasets with a different purpose in mind. For instance, two recent studies described new PTMs after reanalyzing available phosphoproteomics studies [123,124]. Some proteogenomics studies have also made use of public datasets [125].

Data from repositories is often used in the design of SRM/MRM transitions for targeted proteomics approaches [126]. A mentioned already, GPMDB and PeptideAtlas provide already this functionality. Data from PRIDE is being used by the MRMAID resource, with the same purpose in mind [127]. Another popular data reuse is the building of spectral libraries. Several repositories build their own libraries (e.g., PeptideAtlas, PRIDE) that can be used in spectral searches. In addition, meta-analysis of data in PRIDE (without reprocessing the data) has already happened, enabling the extraction of new knowledge [128–132]. Finally, data in proteomics repositories can also be used to improve the content of the protein sequence databases [133].

In this context, PeptideShaker (http://peptide-shaker.googlecode.com/) is a recently developed Java application [134,135] that allows the postprocessing and visualization of protein identification experiments and it greatly facilitates the reprocessing of PRIDE datasets by using the “PRIDE Reshake” functionality. One of the key features of PeptideShaker is the validation of the results using different values, such as FDR and false-negative rate [136]. These values are displayed in the “FDR/false-negative rate plot” and the availability of these metrics make possible the generation of a “cost/benefit” curve, also known as receiver operating characteristic curve, which enables the users to optimize the quality thresholds.

Finally, it is also important to highlight that not only the publicly available raw data files are used for reanalysis purposes. The availability of output files from pipelines/software can also help third parties to develop new tools and demonstrate its utility. This was the case for the recently developed SpliceVista visualization tool [137].

We strongly encourage proteomics researchers to upload raw data and processed results to public repositories. Before starting a new project, public data can be used to guide new research. This is already common practice in the case of targeted proteomics workflows.

## 5 Pitfalls and future challenges

Data sharing and dissemination is a nontrivial task since it requires substantial investment in infrastructure and software development [138,139]. As mentioned before, one of the aims of the PX consortium is to provide backup when one of the resources has funding problems. As a proof of concept, PRIDE and the defunct Peptidome joined forces to transfer all data from Peptidome to PRIDE [140]. In parallel, as mentioned already, MassIVE has tried to rescue as many datasets originally stored in Tranche as possible, but it has been quite a challenging task.

In June 2012, PRIDE started to accept and handle raw data as part of the PX data workflow. PX and other resources have proven that public data dissemination in a decentralized and well-structured mode is possible and actually important for the proteomics community. In our opinion, the next step would be to foster a higher integration for these resources. At present, the main layer of integration among PX resources happens at the level of the metadata.

The alternative is to access knowledge bases, such as UniProt or neXtProt, but the amount of data coming from MS proteomics repositories in these resources is still limited. Ideally it should be possible for users to look for all available data for a given protein or peptide (including PTMs) in a more straightforward way. At present, scientists need to access the different proteomics resources when they want to get all existing information about a given protein.

**Figure 3:**
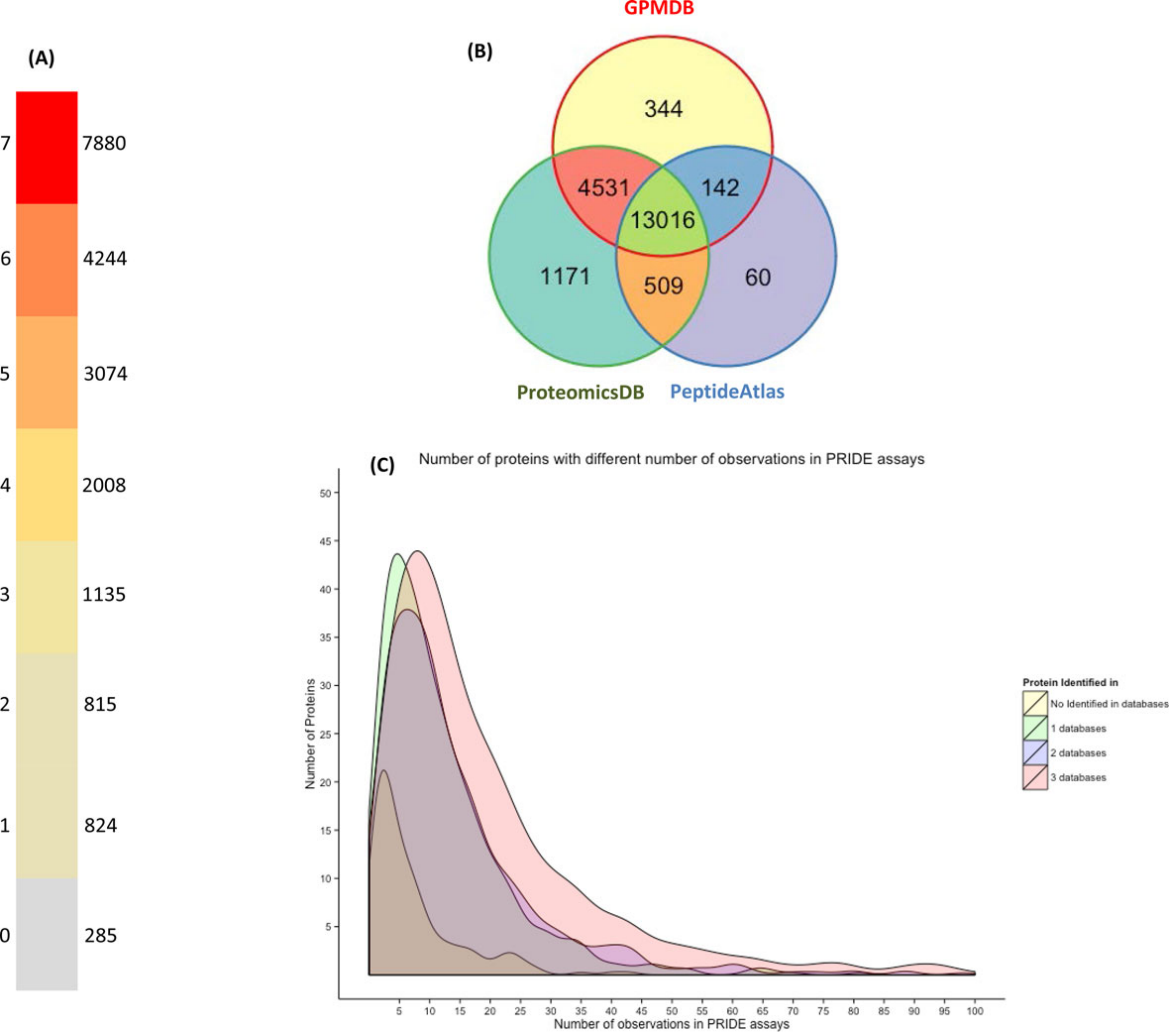
(A) Number of UniProtKB/Swiss-Prot human proteins (release 2014_05, 20,265 entries) observed in different proteomics resources that have a uniform data processing pipeline (GPMDB, ProteomicsDB, PeptideAtlas, HPM, PaxDb, MaxQB, Human Proteinpedia; PRIDE is not included); (B) Venn diagram representing the human protein identifications observed in GPMDB, PeptideAtlas, and ProteomicsDB; (C) Area chart showing the distribution of the number of PRIDE assays for those proteins present in three, two, and one proteomics resources, or for those proteins not identified at all.

To illustrate this idea, we studied the protein expression evidences for the UniProtKB/Swiss-Prot human proteome, including only canonical sequences (release 2014_05, 20,265 entries) in seven resources that have a uniform data processing pipeline: GPMDB, PeptideAtlas, MaxQB, Human Proteinpedia, PaxDb, ProteomicsDB and the HPM. This proved to be a far from trivial task (see all the retrieved data in Supporting Information, including the protein lists taken from each resource). A total of 7880 proteins represent the “core” proteome since they are stored in all the resources (Fig.3A). In addition, 4244, 3074, 2008, 1135, 815 and 824 proteins were identified in six, five, four, three, two and only one resource, respectively (Fig.3A). Only 285 proteins were not found in any of these seven resources. In fact, most of the described resources shared more than 60% of the protein identifications among them (Supporting Information). In our opinion, this redundancy can be used as a “reliability” measure of the evidence of protein expression. For instance, the 13 016 proteins identified in GPMDB, PeptideAtlas, and ProteomicsDB are more likely to be true-positives than the 1171 proteins identified only by ProteomicsDB (Fig.3B). At the same time, the “exclusive” identifications in ProteomicsDB are new valuable evidences, but probably need further investigation.

As mentioned already, the identification of false-positives in big resources and/or when different datasets are combined is still a problem [18,26] and protein expression resources should be as thorough as possible in the statistical analysis. With the identification of a high proportion of the proteins in the human proteome [28,34,63], new analysis tools should be implemented to retrieve protein expression evidences from different resources and to detect/highlight those identifications considered as the most and/or least reliable.

In our opinion it is also needed to increase the reuse and reanalysis of the available data, since they can provide new valuable scientific knowledge. To illustrate this idea, Fig.3C shows the distribution of the number of PRIDE assays for those proteins present in three, two, and one proteomics resources (Fig.3A), or for those proteins not identified at all. It can be observed that, when a protein is identified in three resources, those proteins are likely to be identified, in average, in 13 different PRIDE assays. More interesting is the case of the least observed proteins. For example, if a protein is observed in only one resource, it is observed in nine different PRIDE assays in average. In addition, those 285 proteins without any evidence can be observed in an average of five different PRIDE assays. Therefore, the number of times one given protein is reported in PRIDE (especially if not present in other resources) could be used to select datasets to be reanalyzed.

Finally, another challenge is that at present, studies integrating different “omics” technologies are becoming more and more popular. This type of studies poses a challenge for traditional repositories (which are usually field-specific) and researchers since it is not straightforward to link data from different approaches, for instance MS proteomics and RNAseq data obtained in the same study. Some big institutes such as the EBI and NCBI have implemented specific databases for BioSamples [141] to enable the linking between different studies performed using the same sample. The use of sample identifiers is now starting to facilitate the connection between different “omics” datasets.

## 6 Conclusions

In recent years, there has been a big progress in the development of proteomics repositories and protein expression databases. However, more integration between those databases is required. More tools, such as PRIDE Inspector and PeptideShaker, should be developed to make easier the visualization, reuse, and reanalysis of the data in repositories. Simultaneously, in order to engage users, it seems advisable to monitor download activity of public datasets and measure the community interest on them. Since proteomics has become such a fundamental part of biological research, it is expected that the amount of information available in proteomics resources will keep growing in the coming years. We encourage researchers to make use of this plethora of data.

## Abbreviations

C-HPP: chromosome-based Human Proteome Project
COPaKB: Cardiac Organellar Protein Atlas Knowledgebase
FDR: false discovery rate
HPM: human proteome map
HPRD: human protein reference database
MassIVE: Mass Spectrometry Interactive Virtual Environment
MOPED: Model Organism Protein Expression Database
NCBI: National Center for Biotechnology Information
PASSEL: PeptideAtlas SRM Experiment Library
PSM: peptide spectrum match
PX: ProteomeXchange
TIQAM: targeted identification for quantitative analysis by MRM
TPP: trans-proteomic pipeline
